# Infection-related hospitalizations over 30 years of follow-up in patients starting renal replacement therapy at pediatric age

**DOI:** 10.1007/s00467-015-3209-0

**Published:** 2015-10-13

**Authors:** Danilo Lofaro, Judith L. Vogelzang, Karlijn J van Stralen, Kitty J. Jager, Jaap W. Groothoff

**Affiliations:** Kidney and Transplantation Research Centre, Department of Nephrology, Dialysis and Transplantation, Annunziata Hospital, Via F. Migliori, 87100 Cosenza, Italy; Department of Pediatric Nephrology, Academic Medical Center, Emma Children’s Hospital, Amsterdam, The Netherlands; ERA-EDTA Registry and ESPN/ERA-EDTA Registry, Department of Medical Informatics, Academic Medical Center, University of Amsterdam, Amsterdam, The Netherlands

**Keywords:** Dialysis, Infections, Pyelonephritis, Renal transplantation, Sepsis

## Abstract

**Background:**

Pediatric renal replacement therapy (RRT) patients surviving long-term are at a much higher risk of mortality compared with the age-matched general population. Recently, we demonstrated a transition from cardiovascular disease to infection as the main cause of death in a long-term follow-up study of pediatric RRT. Here, we explore the burden of infections requiring hospitalization over 30 years of follow-up on RRT.

**Methods:**

The cohort comprised all 234 Dutch patients on RRT under 15 years of age between 1972 and1992. We analyzed infection-related hospitalizations during the period 1980–2010. We evaluated the Hospital Admission Rate (HAR) per patient-years (py) and infectious over non-infectious HAR ratio (HARR).

**Results:**

The HAR decreased significantly over time for all patients. The rate of hemodialysis-related infections decreased between 1980 and 1999, but stabilized during 2000–2010, whereas peritoneal dialysis-related infections decreased progressively. Transplantation-related infections did not change, except for urinary tract infections (UTIs), which increased significantly from 3.3/100 py [95%CI 3.2–3.4] in 1980–1989 to 4.4/100 py [4.2–4.5] in 2000–2010 (*p* <0.001). The contribution of infection to HAR increased significantly in transplanted patients (HARR: 1980–1989: 0.25 [0.2–0.3]; 2000–2010: 1.0 [0.79–1.27],* p* <0.001).

**Conclusions:**

Our findings indicate a relative increase in infections requiring hospitalization over time in patients starting RRT during the pediatric age, especially severe UTIs in transplantation. More attention paid to urological abnormalities in cases of recurrent UTI and tailored adjustment of immunosuppression may reduce risk in these patients.

## Introduction

Patients starting renal replacement therapy (RRT) during the pediatric age and surviving long term are at a much higher risk of mortality compared with healthy peers, up to 30 times greater, according to long-term outcome studies. By definition, these data concern historical patients who underwent a different approach compared with current practices. However, although some data show a decline in death rates among end stage renal disease (ESRD) children over time, a recent paper on ESPN/ERA-EDTA data showed a 55-fold increased risk in patients with pediatric ESRD compared with the general population [1–5].

Infection is one of the major causes of mortality and morbidity in patients on RRT, accounting for the 15–23 % of deaths in this population [6–8]. Various factors may contribute to the high rate of life-threatening infections in these patients, such as an impaired immune function as a result of decreased renal function, the open connection of the peritoneal cavity in patients on peritoneal dialysis (PD) and of the central venous system in hemodialysis (HD) patients, and, above all, the use of immunosuppressive therapy in transplanted patients [9–14]. It is of major concern that recent reports show an increasing trend in infection-related mortality, both in dialysis and transplanted patients, and not only in incident patients, but also in patients with a long history of RRT, years after transplantation [15, 16], with an infectious mortality rate increasing from 0.51/100 patient-years (py) before 1989 to 0.82/100 py after 2000 in a Dutch cohort [17]. Infection is one of the most frequent causes of hospitalization in RRT patients [1, 9, 12, 18–23]. In adults it has been associated with a 10 % death rate within 30 days of admission [16]. Still, studies reporting the burden of severe nonfatal infections in long-term RRT patients are lacking.

Our primary aim was therefore to evaluate the burden of infections requiring hospitalization in patients starting RRT during the pediatric age with a 30-year follow-up. Our secondary aim was to analyze the change in burden of severe infections over time using infection-related hospitalization as a marker. Lastly, as hospitalization patterns in the Netherlands have adopted a higher threshold for admission over the last 30 years, we also analyzed the infection/non-infection admission rate ratio over time, to correct for this change in admission policy.

## Subjects and methods

### Data collection

**Fig. 1:**
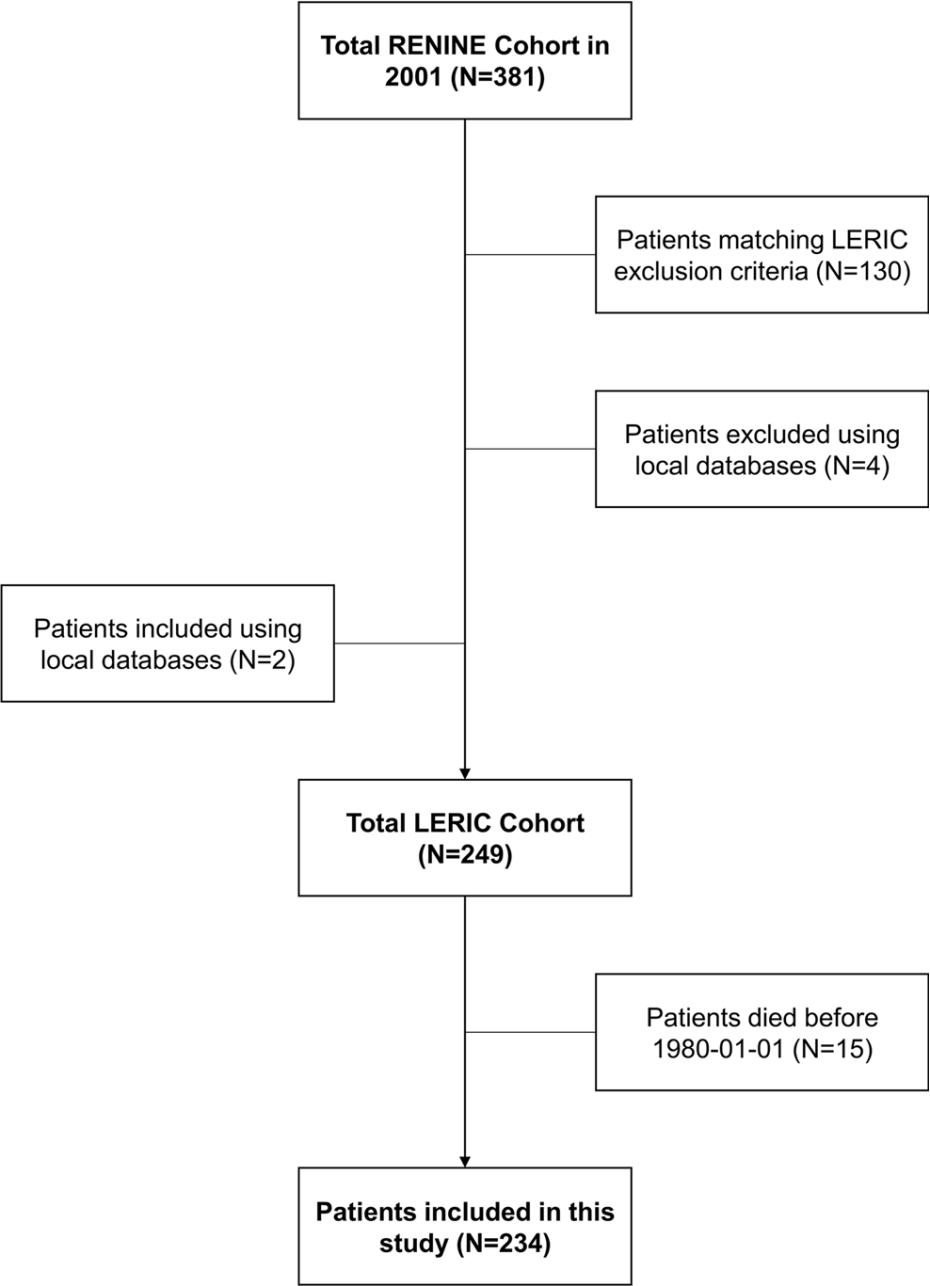
Flow chart of the study cohort. Registry of patients on renal replacement therapy (RENINE), Late Effects of Renal Insufficiency Cohort (LERIC)

We gathered information on patients from the Late Effects of Renal Insufficiency Cohort (LERIC) study, which comprised all Dutch patients who started chronic RRT between 1972 and 1992 at less than 15 years of age, and who were born before 1979. From the original LERIC cohort, we included all patients alive on 1 January 1980 (Fig. 1). Data collection details and results of the first follow-up studies conducted on this cohort between 1998 and 2000 have been described previously [1, 17]. In 2000 and 2010, coworkers from the LERIC study visited all 37 hospitals that had been involved in the medical care of patients during the observation period and collected data on, amongst other things, age at RRT start, start and end dates of HD, PD and transplantation, date and cause of death, and the cause of each hospital admission. In living patients, the day of review was considered to be the end of the observation period.

### Hospital admissions

Information on any hospital admission between 1 January 1980 and the end of the observation period were collected. Causes of hospitalization were classified independently by three reviewers (JLV, JWG, and KJJ), using a detailed description of each patient status around the admission date. Admissions for child birth, dialysis access placement and transplantation were classified as “planned” and excluded from the analyses. Infection-related hospitalizations were classified as: airway infection, gastroenteritis, peritonitis, sepsis, urinary tract infection (UTI), vascular access infection or other infections. Other infections include all infections of unknown origin. Furthermore, bacterial infections were defined as any positive bacterial cultures, abscesses, UTIs, sepsis, tunnel/central venous line infections, PD peritonitis (except when indicated other), sinusitis, segmental pneumonia, bilateral pneumonia with severe disease, pneumonia successfully treated with antibiotics, “fever with chills and antibiotic treatment.” Infections defined as viral included all diagnoses due to a specific virus, gastroenteritis except when indicated as bacterial, and upper airway infection.

### Statistical analysis

In this paper, we compare the rate of hospital admission and infection-related admission during 2000–2010 with the previous two decades (1980–1989 and 1990–1999). The Hospital Admission Rate (HAR) was calculated as the number of hospital admissions per 100 py on RRT with a 95 % confidence interval (95 % CI). Hospital Admission Rate Ratios (HARRs) were calculated to compare infection and non-infection-related admissions. Poisson regression models were used to examine the trends in infection and non-infection HAR with the decade of admission (1980–1989, 1990–1999, and 2000–2010). In the Poisson model, the natural logarithm of the total py at risk was used as the offset. Data analysis was performed using R (v. 3.0.1, R Foundation for Statistical Computing) [24].

## Results

### Study cohort

The total cohort consisted of 234 patients with a median follow-up of 25.0 years (range 0.2–31.4). The median age at the start of RRT was 11.2 years (range 1.9–15.0), and 55.6 % were female. The patient characteristics for each decade are shown in Table 1. Between 2000 and 2010, 2 (1.4 %) patients were lost to follow-up because of emigration, and 1 patient (0.7 %) had avoided medical care since 2007.

**Table 1:** Characteristics of the study population at specific time points during the 30-year follow-up

	1 January 1980 (%)	1 January 1990 (%)	1 January 2000 (%)	1 January 2010 (%)	Total
Number of patients	95	199	185	152	234
Male (%)	39 (41.05)	88 (44.22)	83 (44.86)	71 (46.71)	104 (44.4)
Patients’ age (years)	14.53 (3.60–21.88)	19.48 (11.01–31.88)	29.42 (21.01–41.50)	39.30 (31.01–51.50)	–
CAKUT patients (%)	29 (30.53)	64 (32.16)	62 (33.51)	52 (34.21)	77 (32.91)
Age at RRT (years)	11.00 (1.88– 14.94)	11.25 (1.88–15.87)	11.62 (1.88–21.33)	11.74 (1.88–21.33)	11.39 (1.88–21.33)
Patients on hemodialysis (%)	28 (29.5)	33 (16.6)	23 (12.4)	24 (15.8)	–
Hemodialysis time (years)^a^	1.21 (0.13–6.40)	1.82 (0.03–15.63)	2.23 (0.03–25.63)	2.31 (0.03–35.63)	2.79 (0.03–36.52)
Number of HD					
Patients with at least 1 HD	28 (100)	111 (69.8)	93 (53.4)	90 (49.2)	87 (47.5)
Patients with at least 2 HD	0 (0.0)	35 (22)	47 (27)	50 (27.3)	52 (28.4)
Patients with at least 3 HD	0 (0.0)	11 (6.9)	28 (16.1)	28 (15.3)	29 (15.8)
Patients with more than 3 HD	0 (0.0)	2 (1.3)	6 (3.4)	15 (8.2)	15 (8.2)
Patients on peritoneal dialysis (%)	0 (0.0)	10 (5.0)	18 (9.7)	4 (2.6)	–
Peritoneal dialysis in time (years)^b^	0.04 (0.02–5)	1.03 (0.01–10.73)	1.62 (0.01–15.36)	2.13 (0.01–10.73)	2.42 (0.08–18.55)
Number of PD					
Patients with at least 1 PD	0 (0.0)	47 (92.2)	54 (67.5)	59 (65.6)	62 (66.7)
Patients with at least 2 PD	0 (0.0)	4 (7.8)	22 (27.5)	25 (27.8)	25 (26.9)
Patients with at least 3 PD	0 (0.0)	0 (0.0)	4 (5)	4 (4.4)	4 (4.3)
Patients with more than 3 PD	0 (0.0)	0 (0.0)	0 (0.0)	2 (2.2)	2 (2.2)
Patients on transplantation (%)	67 (70.5)	156 (78.4)	144 (77.8)	124 (81.6)	-
Transplantation time (years)^c^	2.05 (0.01–9.05)	5.77 (0.01–19.06)	13.93 (0.13–29.05)	20.20 (0.01–39.65)	23.43 (0.19–39.06)
Number of TX					
Patients with at least 1 TX	67 (100)	95 (67.9)	104 (57.8)	87 (39.5)	86 (39.1)
Patients with at least 2 TX	0 (0.0)	39 (27.9)	59 (32.8)	83 (37.7)	84 (38.2)
Patients with at least 3 TX	0 (0.0)	6 (4.3)	13 (7.2)	41 (18.6)	41 (18.6)
Patients with more than 3 TX	0 (0.0)	0 (0.0)	4 (2.2)	9 (4.1)	9 (4.1)

*CAKUT* congenital abnormalities of kidney and urinary tract,* RRT* renal replacement therapy,* HD* hemodialysis,* PD* peritoneal dialysis,* TX* transplantation

^a^Patients with at least one period on HD

^b^Patients with at least one period on PD

^c^Patients with at least one period on TX

### All-cause hospital admission rates

**Fig. 2:**
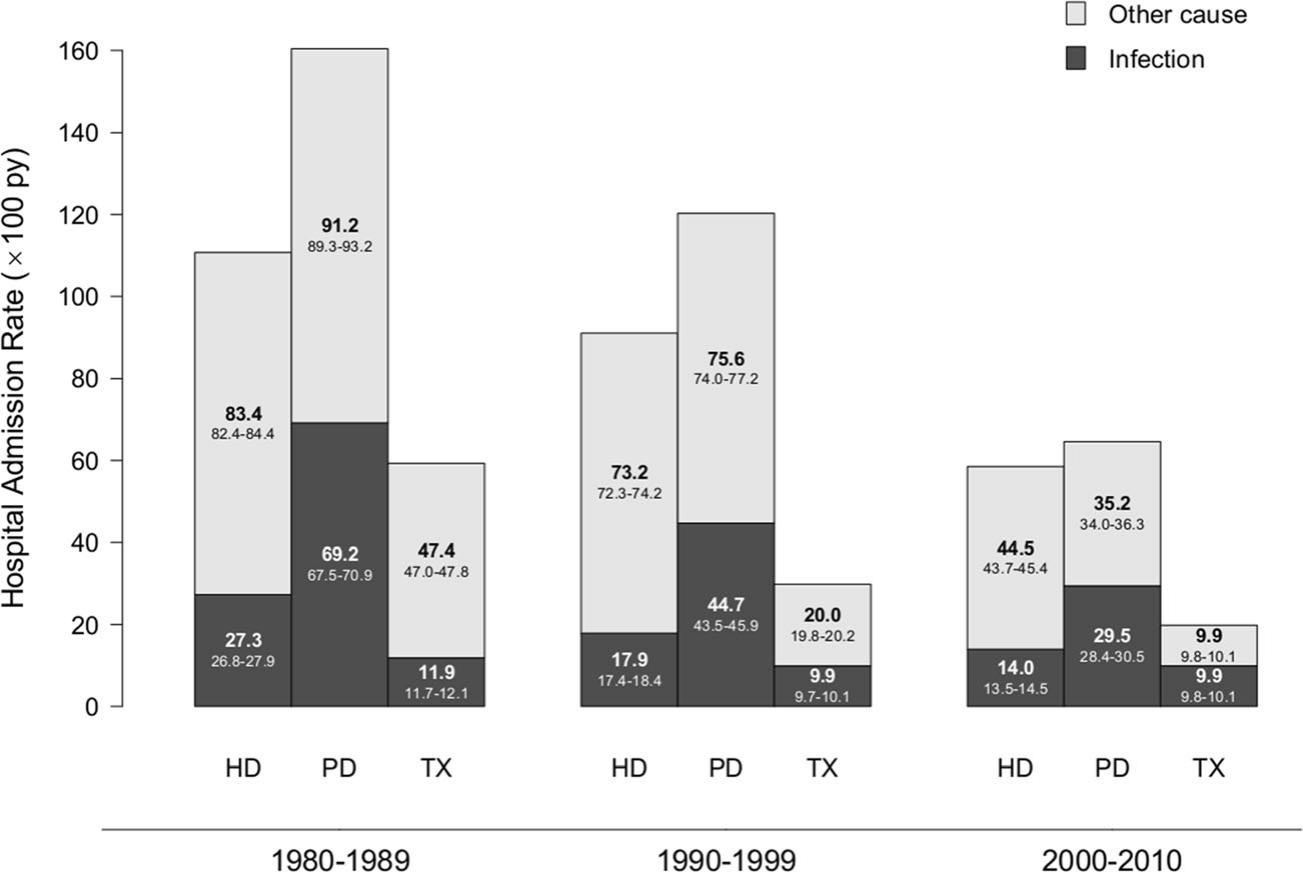
Hospital admission incidence rate for infection and non-infection-related causes by decade and renal replacement therapy (RRT) modality. Inside the bars hospital admission incidence rates with 95 % confidence interval (CI) are presented.* HD* hemodialysis,* PD* peritoneal dialysis,* TX* transplantation

A total of 2,563 hospital admissions were recorded, 757 (29.5 %) of which were infection-related. One thousand two hundred and two admissions occurred between 1980 and 1989, 877 during 1990–1999, and 484 after the turn of the century. Nearly all patients (224 out of 234) experienced at least one unplanned admission during follow-up, with a median of 9 admissions per patient (range 1–100). Over the 30-year period, the average HAR was 48.5/100 py (95 % CI: 48.3–48.7), i.e., almost once every 2 years. HAR was significantly higher in the 1980s (77.0 out of 100 py, 95 % CI: 76.6–77.5,* p* <0.0001) and 1990s (44.6 out of 100 py, 44.3–44.9,* p* <0.0001) compared to 2000–2010 (27.6 out of 100 py, 27.3–27.8). Figure 2 presents the trend in hospital admissions between 1980 and 2010 by decade and RRT modality.

### Infection-related hospital admission rates

Infection-related HAR decreased between 1980–1989 and 1990–1999, but stabilized in 2000–2010. Infection-related HAR in HD patients also decreased from 1980–1989 to 1990–1999, and stabilized after 2000, whereas infection-related HAR in PD patients showed a progressive decrease over time. Conversely, infection-related HAR in transplant patients did not change significantly during the study period, with an almost constant HAR from 11.9 during the 1980s to 9.9 out of 100 py during the 1990s and 2000s.

**Fig. 3:**
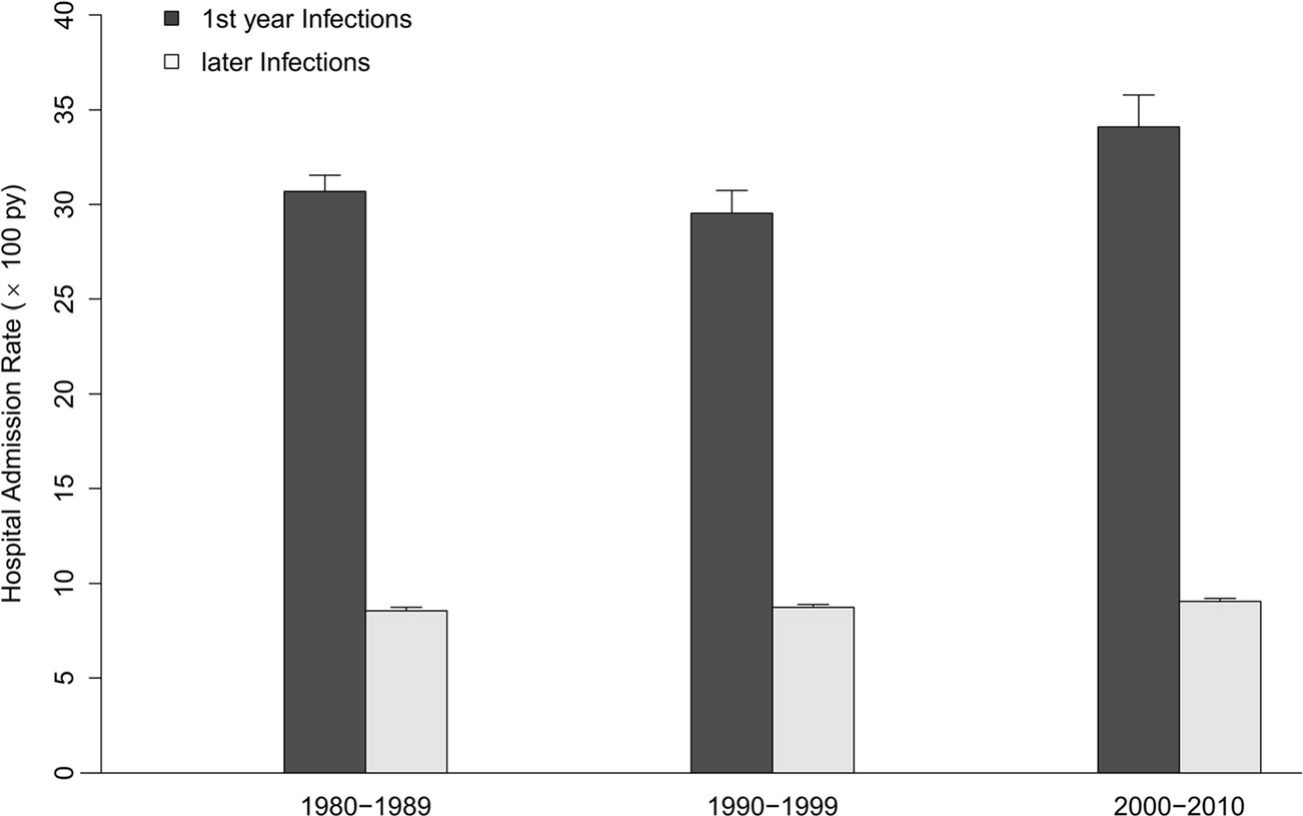
Hospital admissions incidence rate for infection in the first year and later after renal transplantation. Error bars represent 95 % CI

During the first year after transplantation, infection-related HAR was consistently more frequent compared with the period after the first year. Furthermore, there was no improvement over time for either the first-year risk, or the long-term risk of infection-related admission, with a HAR in the 1980s of 30.7 (95 % CI: 29.8–31.5) for the first year vs 8.5 (95 % CI: 8.4–8.7,* p* <0.0001) for later infections and 34.1 (95 % CI: 32.5–35.8) vs 9.0 (95 % CI: 8.9–9.2,* p* <0.0001) during the 2000s (Fig. 3).

### Non-infection-related vs infection-related hospital admission rate ratios

**Fig. 4:**
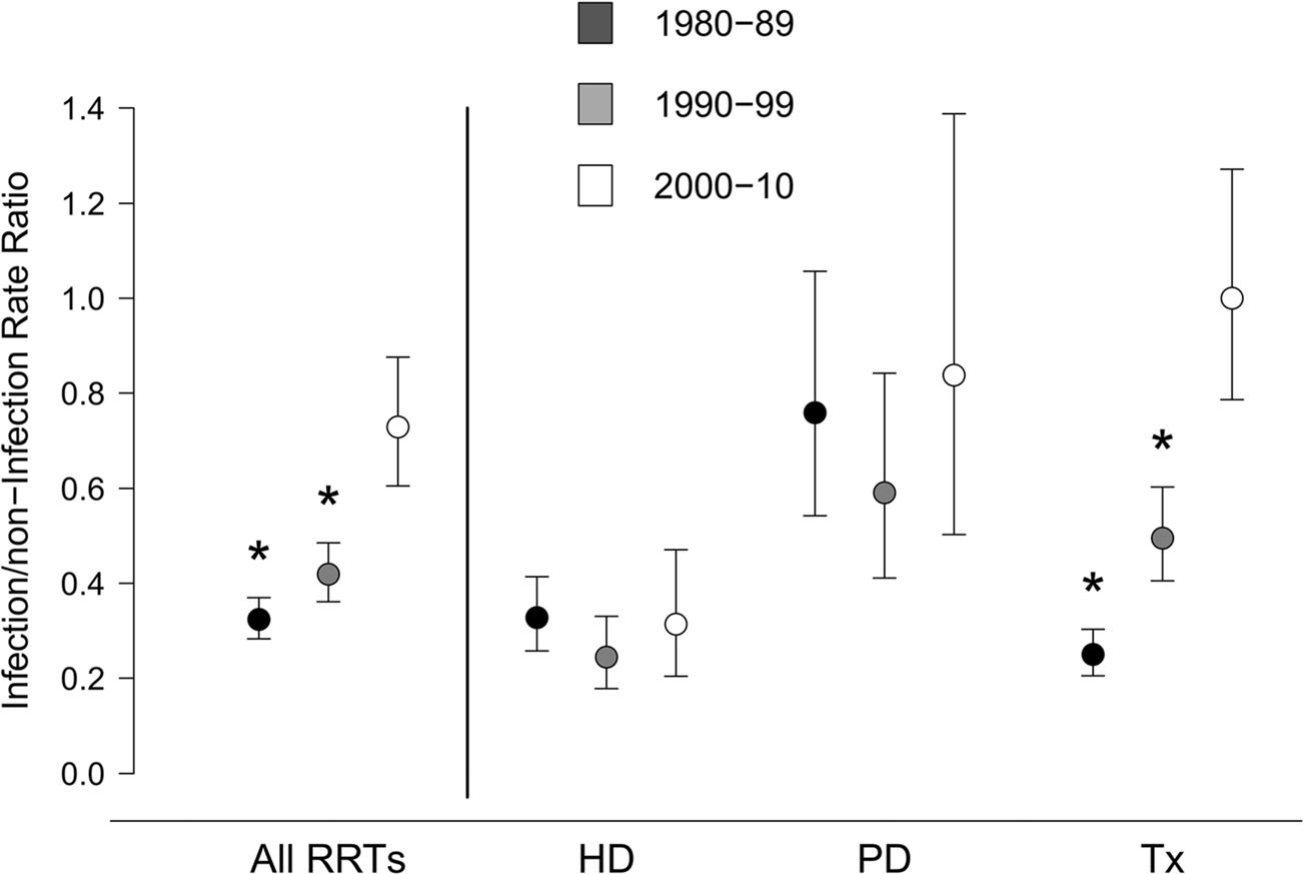
Infection/non-infection Hospital Admission Rate Ratio (HARR) according to decade and RRT modality. *p <0.001 vs the decade 2000–2010. Whiskers represent 95 % CI.* HD* hemodialysis,* PD* peritoneal dialysis,* TX* transplantation

Compared with the rate of admissions for non-infectious causes, the rate of infection-related admissions increased significantly over time: the rate ratio over the 2000s was 0.73 (95 % CI: 0.61–0.88) vs 0.32 (95 % CI: 0.28–0.37,* p* <0.001) and 0.42 (95 % CI: 0.36–0.49,* p* <0.001) for the 1980s and 1990s respectively. This rise was entirely due to a proportional increase in infection-related admissions in transplant patients, with an infection/non-infection ratio rising from 0.25 (95 % CI: 0.20–0.30,* p* <0.001) between 1980 and 1989, to 0.50 (95 % CI: 0.41–0.60,* p* <0.001) between 1990 and 1999, and up to 1 (95 % CI: 0.79–1.27) during the 2000s (Fig. 4).

### Diagnosis-specific, infection-related hospital admission rates

**Fig. 5:**
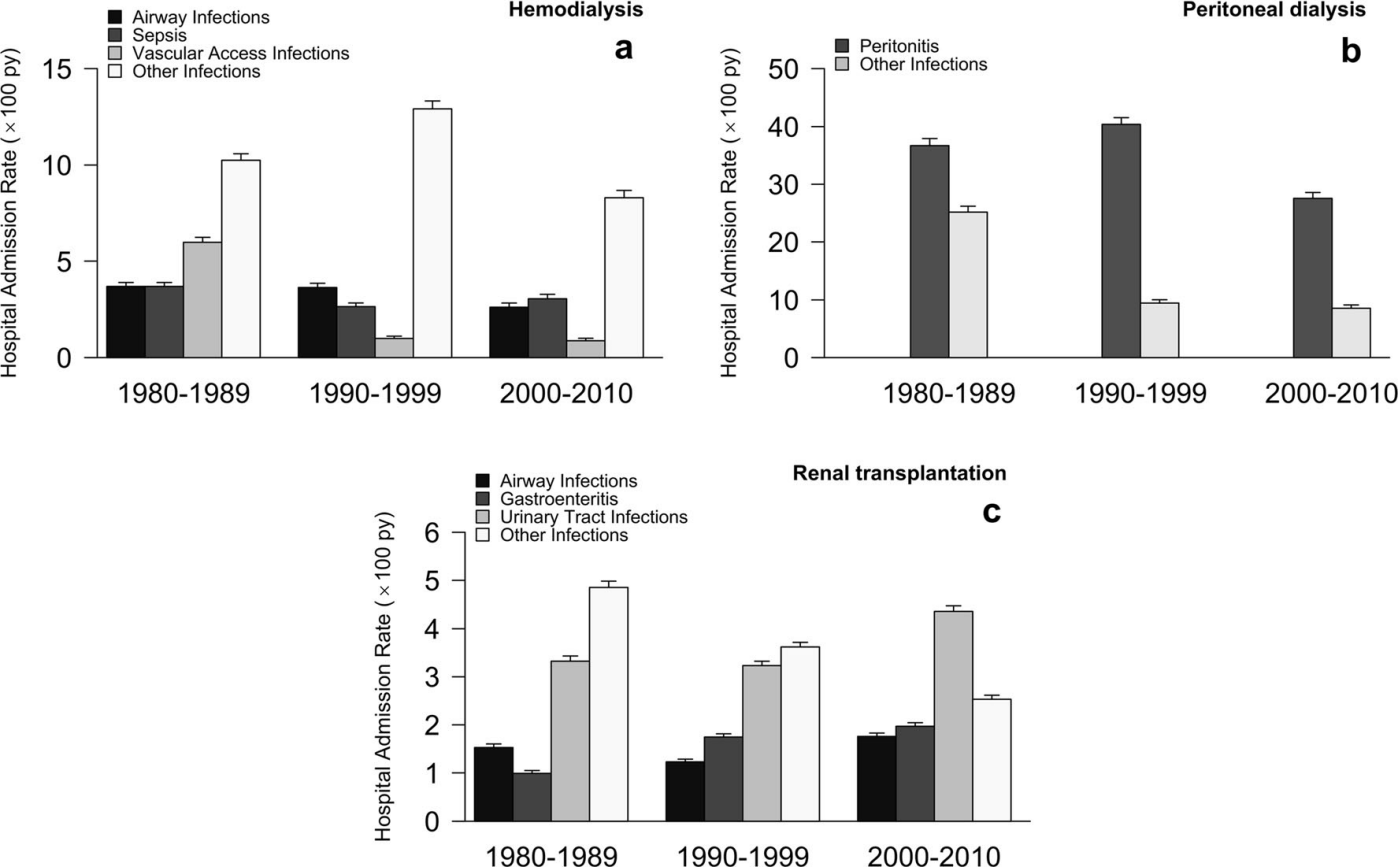
Hospital Admission Rate (HAR) for the most frequent type of infections according to renal replacement therapy (RRT) modality.** a** Hemodialysis;** b** peritoneal dialysis;** c** renal transplantation. Error bars represent 95 % CI

We classified infection-related admissions into specific diagnosis categories: airway infection, gastroenteritis, peritonitis, sepsis, UTI, vascular access infections, and other Infections. Of the 178 HD-related admissions due to infection, 30 (16.8 %) were airway infections, 28 (15.7 %) sepsis, 26 (14.6 %) vascular access infections, and 94 were other types of infection. Rates of airway infection, sepsis, and other infections did not show any significant trend between 1980 and 2010; however, nearly all vascular access infections (21, 11.8 %) occurred during the 1980s (Fig. 5a).

Of the 155 PD-related admissions due to infection, peritonitis was by far the most common cause of admission (111, 71.6 %). The peritonitis-specific admission rate remained constant throughout the follow-up period (Fig. 5b); however, we found a significant increase in peritonitis/other infection ratio from the 1980s (1.4, 95 % CI: 1.3–1.5) to the later decades (3.2, 95 % CI: 3.0–3.5 in the 2000s).

In the 422 transplant-related patients admitted because of infection, the most common forms were UTI (149, 35.3 %), airway infection (61, 14.5 %), and gastroenteritis (66, 15.6 %). In particular, the admission rate due to UTI showed an increase over the decades (Fig. 5c). The majority of UTIs occurred in transplant patients affected by congenital abnormalities of the kidney and urinary tract (CAKUT; HAR was 4.8 out of 100 py, 95 %CI: 4.6–5.0; 3.9 out of 100 py, 95 % CI: 3.7–4.1, and 5.6 out of 100 py, 95 % CI: 5.3–5.8 during the three study decades respectively), which was higher compared with patients with other renal diseases (HAR 2.6 out of 100 py, 95 % CI: 2.5–2.7; 2.9 out of 100 py, 95 % CI: 2.8–3.0, and 3.8 out of 100 py, 95 % CI: 3.6–3.9 in the three study decades respectively,* p* = 0.014). This increase was not due to an increase in the proportion of CAKUT patients over time, as shown in Table 1. In these patients, UTI in the first year after transplantation was significantly more frequent than later in the 2000s (HAR 18.1 out of 100 py, 95 % CI: 16.9–19.3 vs 3.6 out of 100 py, 95 % CI: 3.5–3.8,* p* <0.001), and in the 1980s (HAR 7.8 out of 100 py, 95 % CI: 7.4–8.3 vs 2.7 out of 100 py, 95 % CI: 2.6–2.9,* p* = 0.005), but not in the 1990s. Furthermore, as expected, we found a large difference between the rate of UTI in male (HAR 8.5 out of 100 py, 95 % CI: 8.2–8.8; 6.0 out of 100 py, 95 % CI: 5.8–6.2, and 9.8 out of 100 py, 95 % CI: 9.5–10.1 in the three study decades respectively) and female patients (HAR 0.7 out of 100 py, 95 % CI: 0.6–0.8; 1.8 out of 100 py, 95 % CI: 1.7–1.9, and 1.7 out of 100 py, 95 % CI: 1.6–1.8 in the three study decades respectively,* p* <0.01), and this difference was significant both for CAKUT (*p *= 0.005) and for non-CAKUT patients (*p *= 0.02).

Of the 757 infection-related admissions recorded during the study period, 482 (63.7 %) were classified as bacterial, 163 (21.5 %) as viral, and 15 (1.9 %) as fungal or due to mycoplasma, while 95 (12.5 %) were of unknown origin. We found no significant trends in bacterial infections (changing from a HAR of 5.9 out of 100 py, 95 % CI: 5.8–6.1 in the 1980s to 6.8 out of 100 py, 95 % CI: 6.6–6.9 in the 2000s,* p* = 0.213), whereas the number of viral infections significantly decreased over time, from 3.7 out of 100 py, 95 % CI: 3.6–3.8 in the 1980s to 2.9 out of 100 py, 95 % CI: 2.8–3.1 in the 2000s (*p* = 0.021).

## Discussion

We analyzed the burden of severe infections over 30 years of RRT in patients with pediatric onset of RRT using hospitalizations as a marker. We found a consistent increase in the infection/non-infection HARR, suggesting an increase in the relative contribution of infections to hospital admissions over the past decades. This increase was exclusively found in patients living with a functioning renal graft, where UTIs accounted for the majority of admissions.

Very few data exist on the long-term effects of RRT, as most studies report on data covering much shorter periods of observation. Our data show a high burden of transplant-related infections, not only during the first year after transplantation, as previously described by others [25–28], but also long afterward. We found no significant changes in the first year/later infection ratio throughout the observation period. This is especially remarkable as these patients were on average between 30 and 40 years old in 2000–2010, an age that is associated with the lowest risk of death by infection according to most RRT registry studies [29, 30]. Our findings, therefore, most probably reflect a more general trend toward a relative increase in infections in renal transplant patients over the last 10 years. This trend is in line with the shift from cardiovascular to infectious disease as the primary cause of death, on which we previously reported [17], as well as with reports on admission rates for infections during the first post-transplant year [15, 25, 29, 31, 32].

Infections, even if not lethal, are especially worrying in transplantation patients as they may lead to graft loss. UNOS data have shown an increase in death-censored graft failures due to infections between 1997 and 2006 from 6.4 to 10.1 % [33]. Infection may lead to graft failure in several ways. It may activate the immune system and trigger cytokines that may induce interstitial inflammation, leading to chronic allograft nephropathy. BK and CMV viruses may cause tubulo-interstitial nephritis and bacterial pyelonephritis, and may also directly damage the renal graft [21]. According to the UNOS data, UTIs related to urological complications were associated with an 8.8-times increased risk of death-censored graft failure [33].

The most plausible cause for the increase in burden of transplant-related infections is the concurrent tendency toward the use of more potent immunosuppressive strategies in renal transplantation over the last 20 years [34]. There is abundant evidence for a direct relationship between the extent of immunosuppression after transplantation and the risk of infection [31, 35, 36]. It is not the specific type of drug, but the use of higher dosages, especially of calcineurin inhibitors, and the use of triple instead of double therapy, that have been found to be associated with both a decrease in acute rejections and, as a tradeoff, more infections, such as polyomavirus and UTI [37–40]. In the Netherlands, and similarly in many other countries, all centers for adult renal transplantation introduced IL-2 blockers during the early 2000s as part of induction therapy, and all centers have switched from cyclosporine to high-dose tacrolimus in combination with mycophenolate mofetil as part of induction therapy. Indeed, this implies a substantial increase in the average immunosuppressive dose over the last 10 years [34]. A Spanish observational study showed that basiliximab as part of induction therapy was associated with UTI after renal transplantation [28]. The observed rise in hospital admissions related to UTI in transplantation patients may also be due to the increasing use of antibiotic prophylaxis after renal transplantation, causing a rise in multi-resistant infections that frequently require intravenous antibiotic treatment and hospitalization [40, 41]. In addition, a general increase in the incidence of infections in Western countries has been reported, possibly due to several factors, such as an increase in multi-resistant infection incidence and improved detection and reporting practices [35, 42, 43].

For PD, we observed a gradual decrease over time in infection-related admissions, except for peritonitis. The persisting burden of PD-related peritonitis contrasts with data from two European centers, an Australian center and a Canadian Registry, all showing a decreasing trend over time [44–47]. An explanation might be that these studies analyzed different patients in different eras in contrast to ours, where we followed one closed cohort of patients over time. In these studies, profiles of patients within the different eras did not vary significantly in duration of RRT and, consequently, co-morbidity, whereas in our study, patients who were on PD in the decade 2000–2010 had, by definition, more years of RRT and a greater dialysis burden than in the years before. Conversely, those surviving to 2000–2010 may be considered less susceptible to various peritonitis risk factors as they can be considered “survivors”. Therefore, this finding raises concern, especially as it is also at odds with the major reduction in vascular access-related infections seen in HD over time.

This study has several limitations. First, we used hospitalization as a marker for the burden of severe infections. Hospitalization has been used as an indicator of disease severity for various other diseases, as severity assessment scores may have even more limitations [48–50]. Changes in hospitalization rates over time are fraught with difficulties because they may be subject to underlying trends in patterns of care. A change in the rate of hospitalizations not only reflects disease severity or incidence, but also organizational issues, such as improved methods of outpatient care, a change in the diagnostic criteria for infections, and changes in nephrologist hospitalization and treatment policies from the 1980s up to the present day. In Europe, there is a general trend toward lower numbers and shorter hospital stays. This is clearly reflected in the decreasing number of non-infectious-related hospitalizations. Therefore, the relatively constant rate of infectious hospitalizations may even be flawed, as in reality there may have been a sharp increase in the number of infections, but patients were not admitted to hospital. Another limitation is the retrospective collection of the data, which in some cases hampered an accurate distinction between bacterial and other causes of infection. Finally, following a closed cohort of patients over such a long period, patients surviving up to 2000 after a long history of RRT may represent a very specific population that may be difficult to compare with all prevalent RRT patients. For instance, patients returning to dialysis after transplant failure could be at a higher risk of infection than naïve dialysis patients.

In short, we found evidence for a significant increase in the burden of clinically significant infections in transplanted patients over the past decades, not only in the first year after transplantation, but also among patients who have been living with a functioning graft for a prolonged period of time. This high risk of infection should be taken into account in the management of patients with a long history of RRT, such as a more tailored down immunosuppressive regimen in patients with no history of rejection and the specific analysis of urological function in patients with recurrent UTI.

## References

[1] Groothoff JW, Gruppen MP, Offringa M, Hutten J, Lilien MR, van de Kar NJ, Wolff ED, Davin JC, Heymans HS (2002). Mortality and causes of death of end-stage renal disease in children: a Dutch cohort study. Kidney Int.

[2] McDonald SP, Craig JC (2004). Long-term survival of children with end-stage renal disease. N Engl J Med.

[3] Samuel SM, Tonelli MA, Foster BJ, Alexander RT, Nettel-Aguirre A, Soo A, Hemmelgarn BR (2011). Survival in pediatric dialysis and transplant patients. Clin J Am Soc Nephrol.

[4] Chavers BM, Molony JT, Solid CA, Rheault MN, Collins AJ (2015). One-year mortality rates in US children with end-stage renal disease. Am J Nephrol.

[5] Chesnaye N, Bonthuis M, Schaefer F, Groothoff J, Verrina E, Heaf J, Jankauskiene A, Lukosiene V, Molchanova E, Mota C, Peco-Antić A, Ratsch I-M, Bjerre A, Roussinov D, Sukalo A, Topaloglu R, Van Hoeck K, Zagozdzon I, Jager K, Van Stralen K (2014). Demographics of paediatric renal replacement therapy in Europe: a report of the ESPN/ERA–EDTA registry. Pediatr Nephrol.

[6] de Jager DJ, Grootendorst DC, Jager KJ, van Dijk PC, Tomas LM, Ansell D, Collart F, Finne P, Heaf JG, De MJ, Wetzels JF, Rosendaal FR, Dekker FW (2009). Cardiovascular and noncardiovascular mortality among patients starting dialysis. JAMA.

[7] Steenkamp R, Shaw C, Feest T (2013). UK Renal Registry 15th annual report: Chapter 5 survival and causes of death of UK adult patients on renal replacement therapy in 2011: national and centre-specific analyses. Nephron Clin Pract.

[8] Bouwcollege (CBZ) (2003) Developments in the use of hospital beds. I. (Ontwikkelingen bedgebruik ziekenhuizen, deel 1). Utrecht: National Board for Hospital Facilities, in Dutch, English summary

[9] Chavers BM, Solid CA, Gilbertson DT, Collins AJ (2007). Infection-related hospitalization rates in pediatric versus adult patients with end-stage renal disease in the United States. J Am Soc Nephrol.

[10] Aslam N, Bernardini J, Fried L, Burr R, Piraino B (2006). Comparison of infectious complications between incident hemodialysis and peritoneal dialysis patients. Clin J Am Soc Nephrol.

[11] Comoli P, Ginevri F (2012). Monitoring and managing viral infections in pediatric renal transplant recipients. Pediatr Nephrol.

[12] Mitsnefes MM, Laskin BL, Dahhou M, Zhang X, Foster BJ (2013). Mortality risk among children initially treated with dialysis for end-stage kidney disease, 1990–2010. JAMA.

[13] Pastan S, Soucie JM, McClellan WM (2002). Vascular access and increased risk of death among hemodialysis patients. Kidney Int.

[14] Vanholder R, Van Biesen W (2002). Incidence of infectious morbidity and mortality in dialysis patients. Blood Purif.

[15] Dharnidharka VR, Stablein DM, Harmon WE (2004). Post-transplant infections now exceed acute rejection as cause for hospitalization: a report of the NAPRTCS. Am J Transplant.

[16] Chopra I, Hesse L, O'Neill A, van der Goot H (2002). Discovery and development of new anti-bacterial drugs. Trends in drug research III.

[17] Vogelzang JL, van Stralen KJ, Jager KJ, Groothoff JW (2013). Trend from cardiovascular to non-cardiovascular late mortality in patients with renal replacement therapy since childhood. Nephrol Dial Transplant.

[18] North American Pediatric Renal Trials and Collaborative Studies (2010) NAPRTCS 2010 annual transplantation report. Available at: https://web.emmes.com/study/ped/annlrept/2010_Report.pdf

[19] North American Pediatric Renal Trials and Collaborative Studies (2011) NAPRTCS 2011 annual dialysis report. Available at: https://web.emmes.com/study/ped/annlrept/annualrept2011.pdf

[20] Allon M, Depner TA, Radeva M, Bailey J, Beddhu S, Butterly D, Coyne DW, Gassman JJ, Kaufman AM, Kaysen GA, Lewis JA, Schwab SJ (2003). Impact of dialysis dose and membrane on infection-related hospitalization and death: results of the HEMO Study. J Am Soc Nephrol.

[21] Dupont PJ, Manuel O, Pascual M (2010) Infection and chronic allograft dysfunction. Kidney Int Suppl (119)S47–S53 10.1038/ki.2010.423 21116318

[22] Johnson DW, Dent H, Hawley CM, McDonald SP, Rosman JB, Brown FG, Bannister KM, Wiggins KJ (2009). Associations of dialysis modality and infectious mortality in incident dialysis patients in Australia and New Zealand. Am J Kidney Dis.

[23] Rayner HC, Pisoni RL, Bommer J, Canaud B, Hecking E, Locatelli F, Piera L, Bragg-Gresham JL, Feldman HI, Goodkin DA, Gillespie B, Wolfe RA, Held PJ, Port FK (2004). Mortality and hospitalization in haemodialysis patients in five European countries: results from the Dialysis Outcomes and Practice Patterns Study (DOPPS). Nephrol Dial Transplant.

[24] Development Core Team R (2011) R: a language and environment for statistical computing. R Foundation for Statistical Computing, Vienna, Austria

[25] Kosmadakis G, Daikos GL, Pavlopoulou ID, Gobou A, Kostakis A, Tzanatou-Exarchou H, Boletis JN (2013). Infectious complications in the first year post renal transplantation. Transplant Proc.

[26] Alangaden GJ, Thyagarajan R, Gruber SA, Morawski K, Garnick J, El-Amm JM, West MS, Sillix DH, Chandrasekar PH, Haririan A (2006). Infectious complications after kidney transplantation: current epidemiology and associated risk factors. Clin Transplant.

[27] United States Renal Data System (2012) Transplantation. USRDS 2012 annual data report: atlas of end-stage renal disease in the United States. National Institutes of Health, National Institute of Diabetes and Digestive and Kidney Diseases, Bethesda, MD, p 290. Available from http://www.usrds.org

[28] Galindo Sacristan P, Perez Marfil A, Osorio Moratalla JM, de Gracia Guindo C, Ruiz Fuentes C, Castilla Barbosa YA, Garcia Jimenez B, de Teresa Alguacil J, Barroso Martin FJ, Osuna Ortega A (2013). Predictive factors of infection in the first year after kidney transplantation. Transplant Proc.

[29] Pruthi R, Casula A, MacPhee I (2013). UK Renal Registry 15th annual report: demographic and biochemistry profile of kidney transplant recipients in the UK in 2011: national and centre-specific analyses. Nephron Clin Pract.

[30] United States Renal Data System (2013) Transplantation. USRDS 2013 annual data report: atlas of end-stage renal disease in the United States. National Institutes of Health, National Institute of Diabetes and Digestive and Kidney Diseases, Bethesda, MD. Available from http://www.usrds.org

[31] Ekberg H, Tedesco-Silva H, Demirbas A, Vitko S, Nashan B, Gurkan A, Margreiter R, Hugo C, Grinyo JM, Frei U, Vanrenterghem Y, Daloze P, Halloran PF (2007). Reduced exposure to calcineurin inhibitors in renal transplantation. N Engl J Med.

[32] United States Renal Data System (2007) Transplantation. USRDS 2007 annual data report: atlas of end-stage renal disease in the United States. National Institutes of Health, National Institute of Diabetes and Digestive and Kidney Diseases, Bethesda, MD. Available from http://www.usrds.org

[33] Parasuraman R, Abouljoud M, Jacobsen G, Reddy G, Koffron A, Venkat KK (2011). Increasing trend in infection-related death-censored graft failure in renal transplantation. Transplantation.

[34] Kho M, Cransberg K, Weimar W, van Gelder T (2011). Current immunosuppressive treatment after kidney transplantation. Expert Opin Pharmacother.

[35] Nashan B, Curtis J, Ponticelli C, Mourad G, Jaffe J, Haas T (2004). Everolimus and reduced-exposure cyclosporine in de novo renal-transplant recipients: a three-year phase II, randomized, multicenter, open-label study. Transplantation.

[36] Vitko S, Wlodarczyk Z, Kyllonen L, Czajkowski Z, Margreiter R, Backman L, Perner F, Rigotti P, Jaques B, Abramowicz D, Kessler M, Sanchez-Plumed J, Rostaing L, Rodger RS, Donati D, Vanrenterghem Y (2006). Tacrolimus combined with two different dosages of sirolimus in kidney transplantation: results of a multicenter study. Am J Transplant.

[37] Howell DN, Miller SE (2005). Viral infections in solid organ transplants: hitting a moving target. Microsc Microanal.

[38] Säemann M, Hörl WH (2008). Urinary tract infection in renal transplant recipients. Eur J Clin Invest.

[39] Schold JD, Rehman S, Kayle LK, Magliocca J, Srinivas TR, Meier-Kriesche HU (2009). Treatment for BK virus: incidence, risk factors and outcomes for kidney transplant recipients in the United States. Transpl Int.

[40] Wojciechowski D, Chandran S (2013). Effect of ciprofloxacin combined with sulfamethoxazole-trimethoprim prophylaxis on the incidence of urinary tract infections after kidney transplantation. Transplantation.

[41] Green H, Rahamimov R, Gafter U, Leibovitci L, Paul M (2011). Antibiotic prophylaxis for urinary tract infections in renal transplant recipients: a systematic review and meta-analysis. Transpl Infect Dis.

[42] Taneja C, Zervos M, Haque N, Moore C, Reyes K, Spalding J, Jiang J, Oster G (2009). Trends in US hospital admissions for skin and soft tissue infections. Emerg Infect Dis.

[43] Trotter CL, Stuart JM, George R, Miller E (2008). Increasing hospital admissions for pneumonia, England. Emerg Infect Dis.

[44] Nessim SJ, Bargman JM, Austin PC, Story K, Jassal SV (2009). Impact of age on peritonitis risk in peritoneal dialysis patients: an era effect. Clin J Am Soc Nephrol.

[45] Van Esch S, Krediet RT, Struijk DG (2014) 32 years' experience of peritoneal dialysis-related peritonitis in a university hospital. Perit Dial Int 34:162–170 10.3747/pdi.2013.00275 PMC3968101 24584620

[46] Brown F, Liu WJ, Kotsanas D, Korman TM, Atkins RC (2007). A quarter of a century of adult peritoneal dialysis-related peritonitis at an Australian medical center. Perit Dial Int.

[47] Rocha A, Rodrigues A, Teixeira L, Carvalho MJ, Mendonca D, Cabrita A (2012). Temporal trends in peritonitis rates, microbiology and outcomes: the major clinical complication of peritoneal dialysis. Blood Purif.

[48] Szabo SM, Gooch KL, Bibby MM, Vo PG, Mitchell I, Bradt P, Levy AR (2013). The risk of mortality among young children hospitalized for severe respiratory syncytial virus infection. Paediatr Respir Rev.

[49] Chalmers JD, Rutherford J (2012). Can we use severity assessment tools to increase outpatient management of community-acquired pneumonia?. Eur J Intern Med.

[50] Kvaerner KJ, Austeng ME, Abdelnoor M (2013). Hospitalization for acute otitis media as a useful marker for disease severity. Pediatr Infect Dis J.

